# Emotional responses of veterinary students to first encounters with animal body donors

**DOI:** 10.1002/ase.70291

**Published:** 2026-07-14

**Authors:** S. Regnault, C. Basu, S. Channon

**Affiliations:** ^1^ Department of Life Sciences, Aberystwyth School of Veterinary Sciences Aberystwyth University Aberystwyth UK; ^2^ Department of Comparative Biomedical Sciences, School of Veterinary Medicine University of Surrey Guildford UK; ^3^ Department of Comparative Biomedical Sciences The Royal Veterinary College London UK

**Keywords:** anatomy, body, dissection, feelings, practical, prosection, teaching, vet

## Abstract

Students' first encounters with animal body donors in higher education are a formative and also potentially stressful experience. The complex emotions elicited by body donor use in learning and their effects on professional development have been documented in medical students, but are far less explored in veterinary medical counterparts. In this study, we investigated student emotional responses to animal body donors and the anatomy laboratory in 164 first‐year students from three UK veterinary schools. We found that many students feel positive emotions regarding their first body donor encounter, but that it can be a source of negative emotion, too. In particular, performing dissection and the use of whole, recognisable animals prompted significantly more students to report negative emotions (9% and 13% of participants respectively) compared with other class formats and specimen types (*p* < 0.02). As the first year course progressed, students felt generally positive about encountering body donors. Many students report their feelings surrounding body donor use changed after one (10%) or several classes (35%), whilst others reported that their feelings did not change much (43%). Almost all students felt the use of body donors enhanced their learning, with impacts on academic, practical, and interpersonal benefits cited. Whilst the picture of students' first body donor encounter in veterinary education is generally positive, the findings from this study enable us to suggest interventions that may assist in creating positive, intentional, and inclusive learning environments where animal body donors form an important part of curriculum delivery.

## INTRODUCTION

Dissection and the first encounters with body donors in human medical courses are commonly cited as highly emotional and formative experiences.^1^, ^2^ In many cases, the negative feelings are viewed sociologically as a necessary component (a “rite‐of‐passage”),^3^ ^,^ ^4^ which resolves quickly following the dissection.^1^ In some cases, however, students report severe or persistent symptoms of stress resulting from body‐donor‐based practical classes (about 5% of students^5^).

It is not clear whether veterinary students have similar experiences in their first encounters with animal body donors in the classroom. The first known study to specifically examine this topic^6^ investigated anxiety in 168 veterinary students at CEU Cardenal Herrera University (Valencia, Spain), before and after their first class involving dog body donors. They found most students felt positive before the body donor encounter, and experienced a rapid reduction in anxiety following it (i.e., similar to human health courses). However, the emotional trajectory of students beyond this initial teaching session remains unknown. Additionally, it remains unclear how those findings might apply across a range of curricular and environmental contexts, and whether, given the diversity of species to which veterinary students are exposed, specimen type might influence emotional responses.

Anticipatory/phobic anxiety can be heightened for some students due to the environment of the anatomy laboratory, posing biological and physical risks, meaning students are typically not allowed unfettered access to these spaces (authors' personal observation). Previous studies have also examined the emotions of veterinary students in other contexts, such as first surgical encounter with live animals,^7^ or identifying sources of stress in general.^8^, ^9^, ^10^ Anecdotal observations and student interviews from other studies suggest that dissection of traditional pet species can be emotional or stressful for some students.^11^, ^12^ The strong human‐animal bond has also been indicated to influence emotional responses in other work: In free‐form interviews with 24 graduating veterinary students in the USA, Herzog and colleagues^8^ noted that whilst most students did not self‐identify necropsies [post‐mortem dissection] as a distressing experience retrospectively, it was identified as particularly upsetting or stressful for some students when the animal was known to the student, or if it was a small companion species (e.g., dog or cat, likened to a child by one student interviewed). Whether these feelings are universal and widespread amongst veterinary students, 30 years later, and how prior experience and exposure to animal bodies might influence emotional response in first‐year veterinary students is unclear.

The impact that emotional responses such as stress have on learning can be difficult to interpret, with some types of stress appearing beneficial, whilst others appear to inhibit learning or recall.^13^, ^14^ Low level stress or some degree of anticipatory anxiety may be positive (“eustress”^15^) and constitute part of the reason why experiential learning (like a body‐donor‐based practical class) is interesting and memorable for the student. From a constructivist viewpoint, practical classes allow students to directly explore anatomical structure and function within a facilitated environment to discover knowledge for themselves. However, if body‐donor‐related anxiety and stress inhibit learning, it may disproportionately affect the knowledge and skills acquired in the anatomy laboratory when limited time is available to students,^1^ such as in many modern medical and veterinary anatomy courses.^16^, ^17^, ^18^, ^19^ Documentation of the negative aspects of the dissection experience in the medical field has allowed educators to explore strategies to overcome them, such as reducing unnecessary‐but‐traditional experiences,^20^ or equipping students with skills to process their feelings (where such experiences are a helpful or necessary part of professional growth).^1^ Increasing our understanding of the type and trajectory of emotional responses that veterinary students have when encountering animal bodies will allow veterinary anatomy educators to similarly tailor educational experiences and to consider any emotional support required.

This study aimed to elucidate whether animal body donor encounters are a source of negative emotion or stress for first‐year veterinary students (as it is for medical students). Our study builds upon the work of Terrado et al.^6^ and examines students' emotions over an extended timeframe, across multiple institutions (taking into account variable environments, curriculum, and session designs) and regarding several different species. We aimed to use mixed methodologies, both quantitative and qualitative, to ensure examination of both numerical and statistical trends as well as to gain an understanding of the nuanced emotional responses that students experience. We ask: do students perceive body‐donor‐related stress as helpful (eustress) or harmful (distress)? Since previous studies have identified specific circumstances in which encounters with body donors have been anecdotally reported to be particularly challenging for students, secondary objectives of this study were to identify key factors (e.g., species, type of specimen) and themes associated with distress. Finally, the study aimed to investigate how emotion in veterinary students changes over their first year of study.

We hypothesised that:

1. Encounters with body donors are a source of negative emotion for first‐year veterinary students, but these negative emotions exhibit a trajectory of rapid resolution following the initial encounter period (i.e., within several practical sessions)
2. Negative emotional reactions are more common for certain species, types of specimens, and practical class formats. We anticipate that students will more often report negative feelings for:
  - Dissections (where the students take an active role in cutting into body donors) compared with other types of encounters (prosections, skeletal specimens)
  - Whole recognisable animals compared with isolated body parts or tracts; and for companion animal species (such as dogs, cats, and horses) compared with livestock species
3. Students feel that practical sessions involving body donors improve their educational experience; their emotions regarding body donor encounters are helpful (eustress) rather than harmful (distress)

## METHODS

Data for this study were collected from first‐year veterinary students commencing their studies in 2022 in the United Kingdom, via repeated measures collection as two online survey questionnaires hosted on the Jisc Online Survey platform (https://www.onlinesurveys.ac.uk/). The survey questions are available in the supplementary materials. The first survey consisted of an initial questionnaire of 20 items, prior to the first practical class of the academic year. The second survey was a follow‐up questionnaire of 24 items delivered approximately 6 months later, once several different types of practical classes had been undertaken. The questions were structured as categorical items, asking participants to select feelings on different aspects of cadaver‐based practical sessions from a list of qualitative options. These options (emotions) were mapped to either positive, neutral, or negative categories (as determined by the study authors; Figure 1). Each categorical item included a free‐response item for optional elaboration. Participation was considered quasi‐voluntary, as surveys were distributed from each institution, and students were invited to complete the survey in their own time. A random prize draw of one £50 online voucher for each survey was offered as an opt‐in incentive.

**FIGURE 1:**
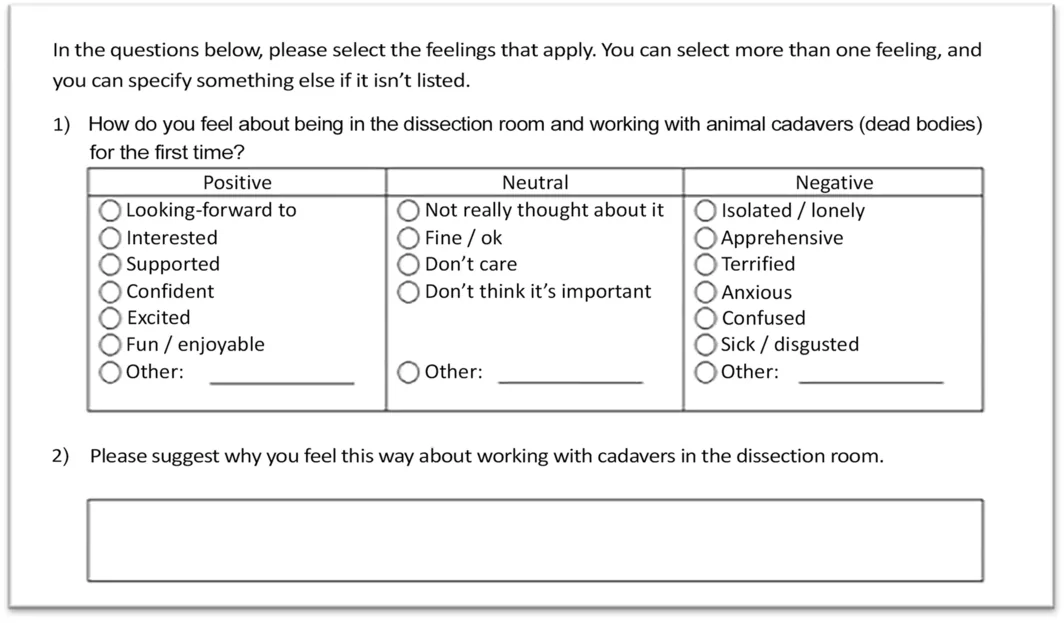
Example question with the list of emotional options presented to respondents in Surveys 1 and 2 (see supplementary material for full survey questionnaire).

First‐year students were recruited from three UK veterinary schools: Aberystwyth School of Veterinary Sciences (ASVS), Royal Veterinary College (RVC; partnered with ASVS), and The School of Veterinary Medicine, University of Surrey (UoS). All three institutions have similar teaching timelines, with a late September/early October start date and several different practical class types involving body donors in the first term of the first year. Study participants were invited to complete each survey via an emailed link to their university email account. In the email and survey, it was emphasised that participation was voluntary and anonymous, and all questions were optional.

The study design was reviewed and received a favourable ethical opinion by Aberystwyth University's Research Ethics and Integrity Officer, Royal Veterinary College Social Sciences Ethical Review Board (URN SR2022‐0141), and University of Surrey's University Ethics Committee (ref FHMS 21‐22 273). The cover letter and any follow‐up emails included signposting to student support services in case students were prompted by the study to consider and process potentially difficult emotions. A named academic from the student's home institution, as well as additional contacts from co‐authors' institutions, were listed, so that potential participants could seek further information from someone known to them (i.e., a lecturer) or someone independent from their institution. A debrief email was also sent to students following initial data analysis, again signposting pastoral support, and as an opportunity to acknowledge that all types of emotional response and all kinds of learners are valid within the Higher Education environment.

The first survey had 164 responses, whilst the follow‐up survey had 102 responses. For Survey 1, the response rate for each participating institution was 11/24 (46%; ASVS), 65/215 (30%; RVC), and 88/173 (51%; UoS). For Survey 2, the response rate was 9/24 (38%; ASVS), 41/215 (19%; RVC), and 52/173 (30%; UoS).

Veterinary Science courses (qualifying students as veterinarians upon graduation) are an undergraduate degree in the UK. There were approximately 2370 students enrolling in Veterinary Science courses in the UK in 2022.^21^ Our study population, therefore, represents about 4%–7% of the UK population of first‐year vet students and is of sufficient size for a 95% confidence interval and 9.5% margin of error for statistical inferences.

In Survey 1, students were asked to select their emotions on a range of scenarios; for example, about the different types of practical sessions involving body donors (called “cadavers” in the survey) that they would encounter in the first year of veterinary courses: dry practical sessions (involving only bones or skeletons), prosections (body donors dissected ahead of time by an academic, ready for students to view); and dissections (students perform the dissection themselves). Subsequent questions presented students with the same options regarding other factors, such as the type and species of specimen that are encountered in class, and the veterinary school teaching environment, including: being in the anatomy laboratory itself, performing dissection tasks (such as cutting and identifying anatomical parts), touching and manipulating animal body donors, smells and sounds that might be encountered, and interactions with teaching staff in the anatomy laboratory. The specific impacts on student learning were explored in a separate survey question, with both positive, negative, and free‐text impacts offered as options.

After several months of teaching, students were offered the opportunity to participate in Survey 2. They were asked to recall how they had felt prior to being in the anatomy lab for their first body donor encounter, and how they now felt regarding various aspects of practical class and body donor encounter, using similar questions as Survey 1. In the second survey, students were also asked to select how they felt about the use of body donors in veterinary education from several options, including the option to enter their own comment. The two surveys were not directly paired, in that responses from specific students are not mapped between the two surveys.

Descriptive statistics were used to characterise the responses. For emotional options, respondents were categorised based on whether they had selected options that were: only positive, mixed positive & neutral, only neutral, mixed positive & negative, mixed negative & neutral, or only negative. Chi‐squared tests were used to test the statistical significance of differences between the proportion of negative‐feeling respondents (i.e., respondents selecting only negative or negative & neutral emotions), comparing types of practical class, types of specimen, and between first and second survey responses. Seventeen chi‐squared comparisons were run (inclusive of post‐hoc testing), and so to account for multiple statistical comparisons and the risk of Type I error, the Benjamini‐Hochberg procedure^22^ was used to control the False Discovery Rate at 5%.

Inductive thematic analysis^23^ was applied to all free‐text comment questions, with the exception of the question seeking information about student prior experience with cadavers. Responses to this question were analysed using content analysis, where the 194 responses (128 from Survey 1 and 66 from Survey 2) were coded into categories, and frequencies of these categories were quantified to allow a proportional representation of prior experiences to be established. Content analysis was more appropriate for this data because the responses were specific, direct, short, and overlapping in nature, and since we wished to gain a semi‐quantified view of prior exposure to animal bodies. Responses provided to this question were not elaborate enough to allow a meaningful thematic analysis of this data.

For the responses that underwent thematic analysis, question responses for all open text questions on Survey 1 (303 comments), as well as equivalent questions on Survey 2 (93 comments) were pooled. A separate thematic analysis was conducted for question 5 on Survey 2 (83 comments) to establish specific reasons why students’ feelings in relation to body donor use changed. Additionally, a separate thematic analysis was used to analyse responses to Survey 2 Q21 (80 comments), which asked students specifically why body donors or the dissection room impacted their learning. The division of data for thematic analysis into these three separate data sets mirrored the main research questions and hypotheses, allowing student emotion to initial exposure, change in emotions, and cadaver impact on student learning to be considered as separate constructs.

Reflexive thematic analysis was used for all analyses of this type.^23^ Data were initially coded in an iterative process, with codes continuously refined and checked as the process of analysis continued. Subsequently, final codes were organised into themes, which were revised and refined in relation to the data and codes. When analysing and interpreting the data, we use critical realism as an ontological standpoint, with a contextualist epistemological approach.

## RESULTS

### Hypothesis 1: Students' initial feelings and the trajectory of resolution

#### Initial emotions

Most first‐year veterinary students who responded to the survey had prior experience with animal cadavers in some capacity; 77% indicated that they had encountered cadavers prior to their first body donor encounter during this study. These previous encounters were primarily during work experience, especially within a small animal general practice environment (32%). Other common encounters took place during work experience on farms (13%), during a previous degree (14%), and at home when students' own pets had died (11%). Notably, relatively few students (7%) encountered cadaver tissue during their school education.

In the first survey, most students reported feeling generally positive about being in the dissection room and working with animal body donors for the first time in their veterinary education (Table 1). The majority of participants selected only positive or a mixture of positive and neutral feelings; a third of respondents selected a mixture of positive and negative feelings; few students selected only neutral feelings, only negative, or a mixture of negative and neutral feelings.

**TABLE 1:** Summary of student emotional responses to animal body donors and changes in response at the two survey time points, Survey 1 and Survey 2.

	Only positive/mixed positive and neutral		Mixed positive and negative		Neutral only		Only negative/mixed negative and neutral
Survey 1	61%		33%		0%		6%
Survey 2: reflection on initial exposure	53%		38%		8%		2%
	**No change—remain positive**	**No change—remain neutral/mixed**	**No change—remain negative**	**Negative/neutral mixed to positive response**	**Negative/mixed to neutral/mixed**	**Positive/mixed/neutral to negative**	**Positive to neutral/mixed**
Survey 2: reported change	44%	16%	0%	30%	1%	2%	7%

Students reported a vast range of underpinning reasons for their reported emotions surrounding practical classes with body donors, which can be represented by five main themes: learning, motivations, sources of anxiety, ethics and values, and background (Figure 2).

**FIGURE 2:**
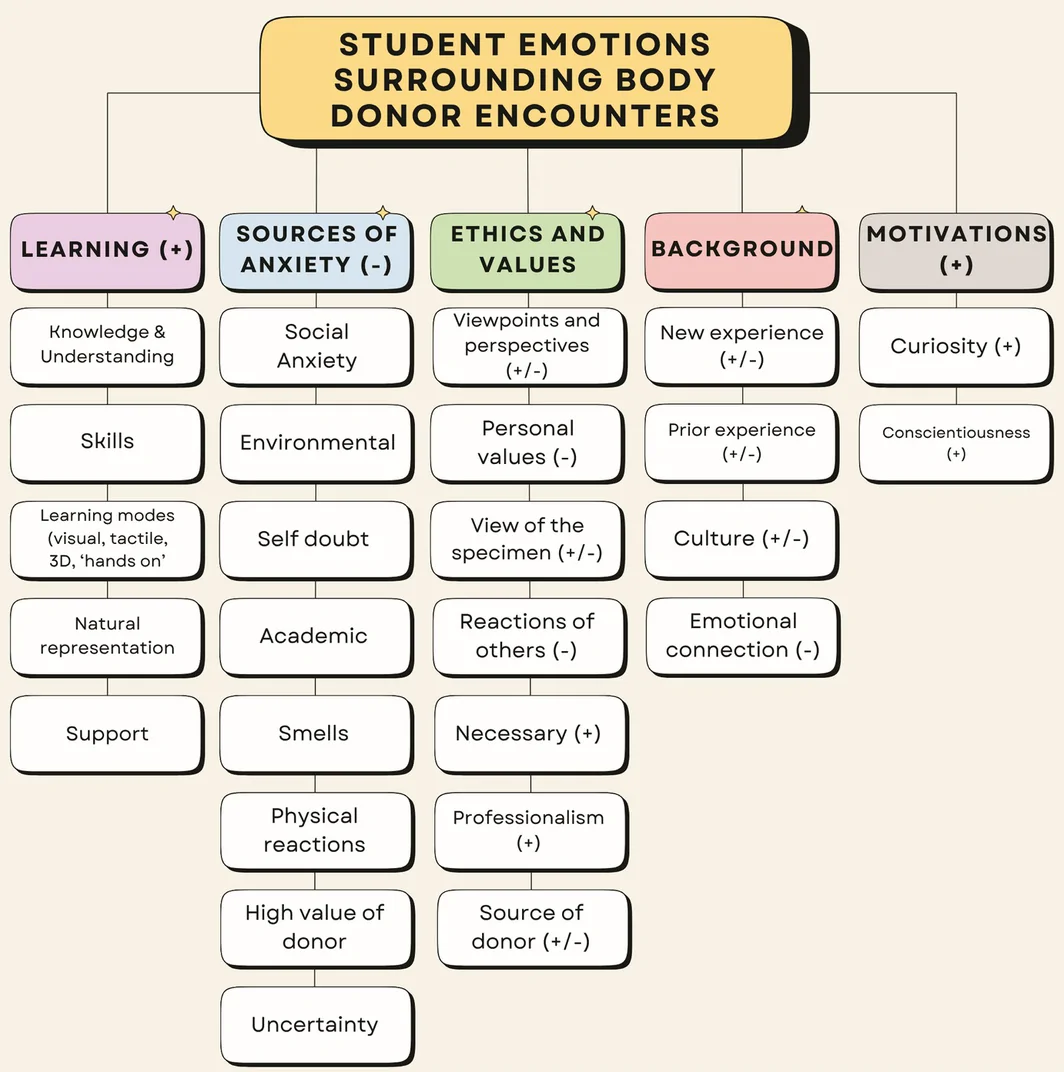
Thematic map outlining themes, and codes within, with respect to underpinning reasons for student emotional responses to body donor encounters.

The theme of learning encompassed reasons underpinning positive or neutral emotions that were exclusively related to perceived positive benefits of practical anatomy teaching using body donors. Perceived learning benefits ranged from those focused on developing knowledge and understanding of the subject matter, through to more applied types of learning, such as the development of skills and the benefit of practical teaching as a learning mode.

I feel it will provide a great source for additional learning and to contextualise the theory learnt in lectures [ID S109]

It will provide a practical view into the theory of muscles and joints and bones we have covered in lectures and allow us to visualise the information [ID S163]

Anatomy is an essential part of our knowledge so seeing the real deal is important [ID S155]

Students commented frequently in regard to the more ‘natural representation’ of body donors, with this ‘real life’ representation being important to them. Some students commented that their emotions prior to encounters with body donors were directly related to the support that staff/their institution had put in place to help with this transition; however, they did not specify what this support was.

Student motivations strongly influenced their outlook ahead of a new experience with body donors, with eagerness to learn more and curiosity about either what the experience might bring or the subject matter frequently given as reasons for a positive outlook.

I am excited to get to explore the bodies of animals that I could be working with when I graduate [ID S145]

…I want to become the best one day and learn as much as I can about the anatomy of animals to understand them [ID S124]

Sources of anxiety were exclusively reported as underpinning negative or neutral emotions. These included more generally an uncertainty about what was to come, but also more specific anxieties about people they might encounter (social anxiety), the new unknown physical environment, academic content, self‐doubt around students' own ability to cope, smells, potential of suffering physical reactions (fainting, sickness), and the high value of the body donor as a learning resource.

Wanting to learn and continue my studies but feel anxiety surrounding the dissection itself—doing it wrong/messing up the specimen/embarrassing myself [ID S1103]

Anxious about possibly fainting or feeling sick and overwhelmed and it being a negative start to the vet course [ID S1105]

I think I am just overall nervous and unsure about what to fully expect in terms of working with cadavers, so the uncertainty adds to the anxiety [ID S120]

Ethics and values strongly influenced student emotions with respect to experiences with body donors. Some students commented on viewing the body donor as a resource or learning tool, and the need to separate the animal from the specimen. Others felt that exposure to dead animal bodies was necessary, and a part of professional life.

I am interested in what we will be doing, but as a vegetarian I am apprehensive on how I might feel whilst doing the practicals [ID S1121]

I view the cadavers as a learning resource and opportunity rather than as someone's pet [ID S1107]

Builds resilience, allows experience from the start. If students are unable to do this then they may not be cut out for the profession [ID S1124]

Some students were concerned about the source or cause of death of body donors, or the reactions of friends or family members. The interaction of ethical frameworks and personal values with other more positive emotions and expectations was acknowledged as a source of complex feelings for students in the lead up to their first experience with body donors.

I am very concerned about where the cadavers are sourced. I assume they are sourced ethically but I would like to be sure [ID S149]

Finally, students' backgrounds were a factor in determining their emotions prior to their first encounter with body donors. Students commented on whether this was a new experience for them, as well as the type and nature of any past cadaver or dissection experiences. Similarly, student cultural background, for example, religion or being from a farming background, was cited as influencing a student's emotional state, as were emotional connections to body donors (for example, drawing parallels with similar species owned as family pets, past emotional events such as the death of a pet, or a family member).

I am anxious about how I will react to a dog cadaver as I am very attached to my own dog [ID S1133]

#### Trajectory of resolution

To understand how individuals' emotions changed over the study period, we asked students participating in Survey 2 to recall how they first felt about being in the dissection room and working with animal body donors for the first time. Most participants recalled feeling generally positive (Table 1; 53%) or a mix of positive and negative for different aspects, rather than neutral or generally negative. These figures broadly reflect the Survey 1 proportions (Table 1). In Survey 2, the majority of students did not report having a significant change in emotional response following several months' experience of practical anatomy teaching (Table 1), either remaining generally positive, generally neutral, or with a mixture of positive and negative emotions for different aspects. No students who said that they were initially negative remained so, and many saw an improvement. However, a small number of students experienced a negative change, from either positive, neutral, or mixed to negative, or from positive to neutral or mixed (Table 1).

When asked how long it took for emotions about any of the types of practical sessions or specimens to change, most students said that their feelings did not change (43%) or that their feelings generally changed after a few classes (35%); fewer said that their feelings changed after one class (10%), within one class (8%), or by the end of the first semester (5%).

Where student emotions or attitudes changed after obtaining experience learning with body donors, students identified a range of underpinning reasons, aligned with the themes of experience and exposure, support, ethics and values, and the learning experience (Figure 3).

**FIGURE 3:**
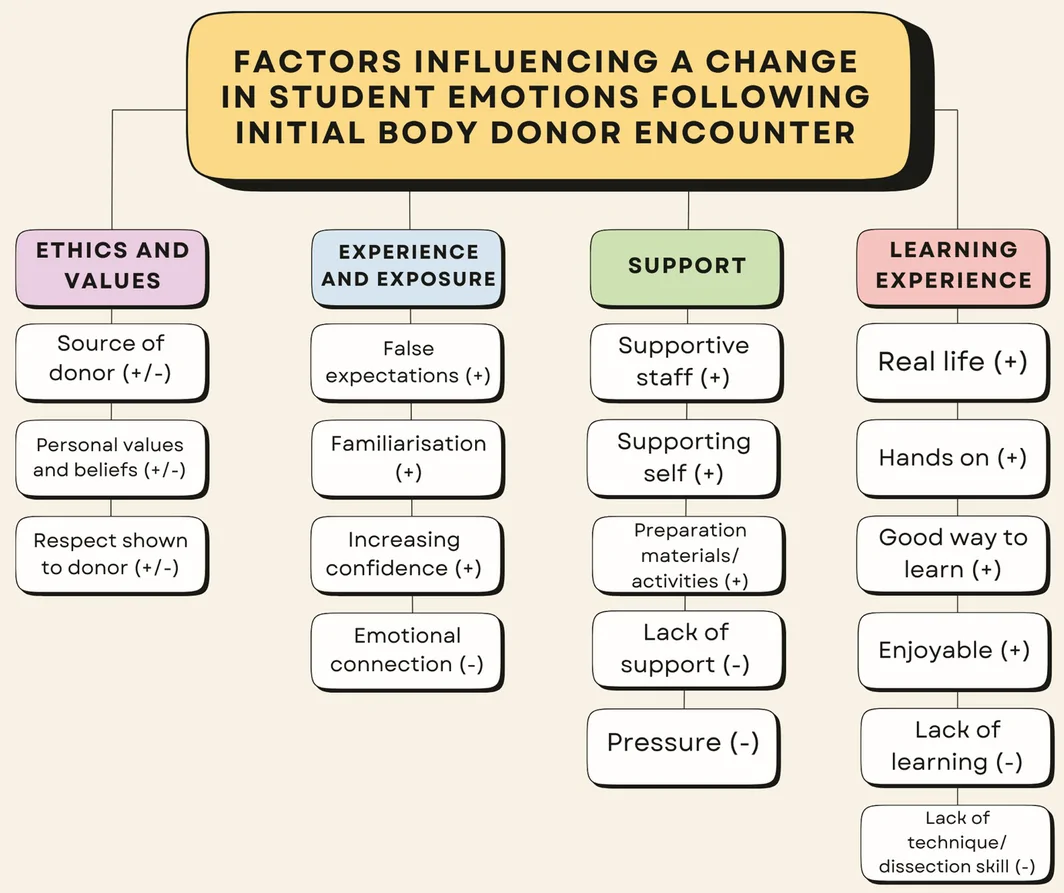
Thematic map outlining themes and codes within, with respect to factors impacting changes in student emotional responses to body donor material.

Students cited that experience and exposure through familiarisation with the teaching spaces, body donors, and smells improved their emotional reaction to the presence of body donors for teaching. They noted that as their confidence increased, so did their emotional well‐being.

I found it a daunting experience to begin with but the more exposure I had to the room and the cadavers the more relaxed I became [ID S218]

Some students felt that their initial concerns were found to be unwarranted after experiencing the teaching sessions.

Once I had realised that it wasn't as bad and disgusting as I thought it might be I felt a lot better [ID S266]

There was a small number of students who felt that exposure to body donors was an unexpectedly unpleasant experience, and in those cases, students reported feeling surprised that past experiences were relived or remembered by taking part in body‐donor‐based teaching.

The first dissection was a rabbit, which was hard for me because I recently lost my pet rabbit of 13 years, so cutting into a dead one was quite confronting [ID S215]

The amount of support from staff was seen to both positively and negatively impact student's initial experiences with body donors. Some students commented that they developed strategies to provide themselves with additional support, which helped them navigate through an experience that they found challenging.

At first I was feeling quite grossed out… I am definitely more used to it and am now properly touching and interacting with the specimens whereas before I would maybe tentatively work myself up to prod something…since the start I've been using positive reinforcement after like treating myself to a long bath or nice food after to make the process feel more rewarding [ID S286]

Students commented that preparatory materials or sessions were helpful when present, and where they were not, there were suggestions from students that these could be beneficial to help prepare students for their first dissection experiences.

I would have liked a talk before our practicals to clear the air about the cadavers and where they are sourced from and why it is important to study using them [ID S215]

Students identified that their personal ethics and values were reasons why their emotions changed following their first body donor exposures. In addition, the amount of respect they felt their colleagues gave to the specimens, and information (including lack of) about the source of animal body material, were also factors that changed their emotional response. All of these factors were seen to both positively or negatively influence student emotions.

I initially got upset about seeing the dead animals but knowing they are being used appropriately and professionally made me feel better [ID S244]

I am apprehensive about where the animals come from… [ID S223]

Many students commented positively on the learning experience with body donors. They cited practicals with body donors as being enjoyable and a ‘good way to learn’ due to the ‘hands on’ nature and ‘real life’ application and relevance.

*As I did more practicals I got used to it and saw how much they helped. I enjoy them now as I can put everything I've learned into a physical context and I often learn more as well*. [ID S219]

Where students were negatively impacted by body donor experiences, this was sometimes linked to their perception that they hadn't learned a great deal, or that their dissection skills were not sufficient to make the most of the learning opportunity. These factors led to a sense of wastefulness of the body donor material.

It was more of an explorative dissection where the students had free reign so I was apprehensive and felt like I was violating the animal and not learning a whole lot [ID S215]

### Hypothesis 2: Responses to encountering body donors within varied classroom contexts

#### Class type

As a proportion, students initially (Survey 1) felt the most positive about dry/skeletal practicals (88%, 1%, and 11% [positive, negative, and neutral/mixed respectively]), followed by prosections (82%, 3%, and 15%), and were least positive about dissections (63%, 9%, 28%; Figure 4). The proportion of respondents initially feeling only negative emotions about dissections was significantly greater than for dry/skeletal practicals (*p* = 0.0003) and for prosections (*p* = 0.01).

**FIGURE 4:**
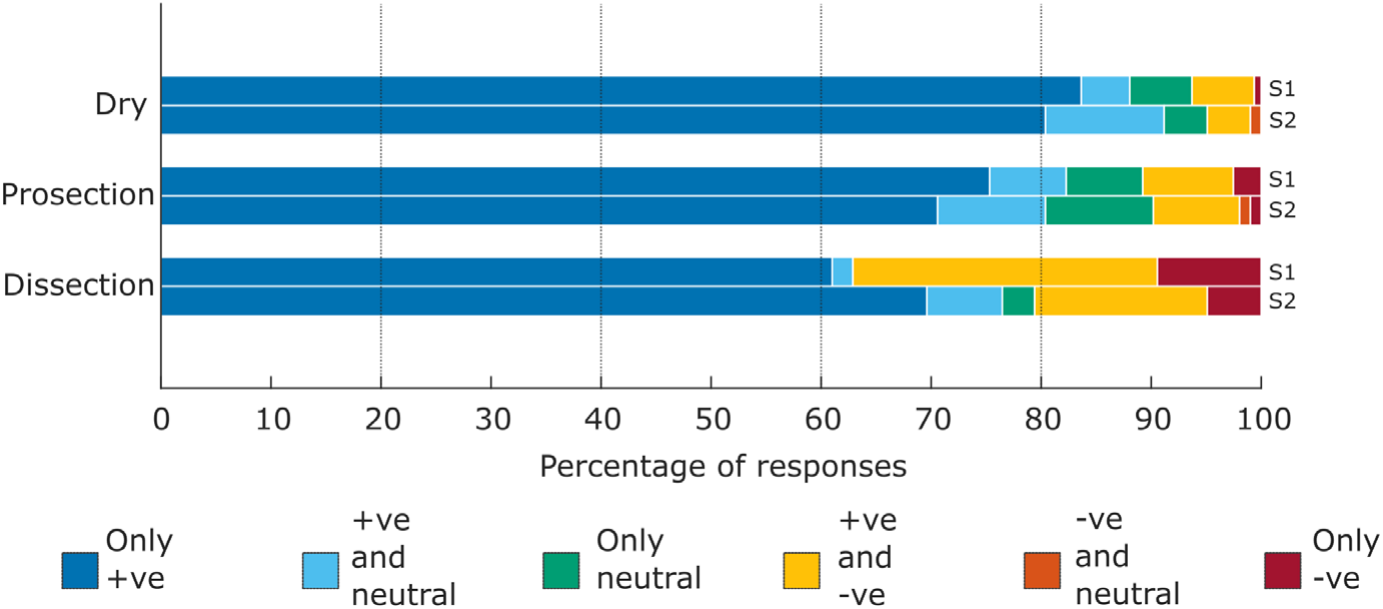
Individual students' selection of emotions by practical class type, for Survey 1 (S1; prior to first practical session) and Survey 2 (S2; after several months' teaching). No statistically significant changes were seen in the proportions of individuals selecting mostly negative feelings between S1 and S2.

Regardless of class type, most of the positive feelings selected by students related to feeling ‘Interested’ and ‘Looking forward to it’. Most of the negative feelings for prosections and dissections were Apprehensive’ and ‘Anxious' (Figure 5).

**FIGURE 5:**
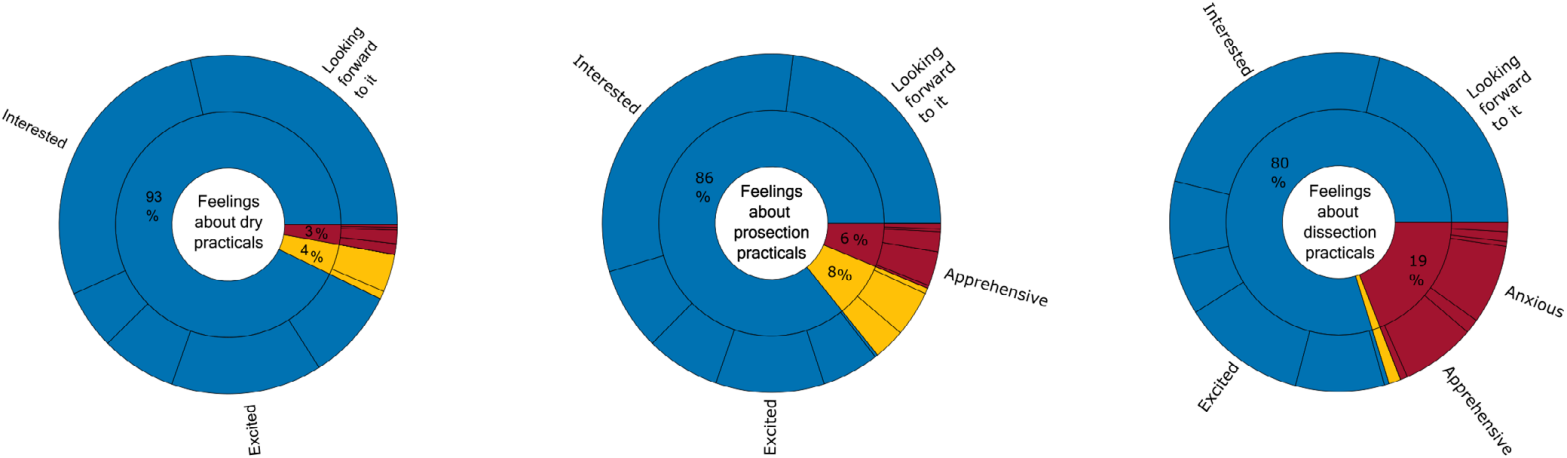
Breakdown of selected feelings by practical class type. Students could select more than one feeling, and the largest proportions are labelled.

With respect to the teaching environment (Figure 6), most respondents were positive about being in the dissection room itself, the idea of performing dissection tasks, touching and manipulating body donors, and interacting with teaching staff. However, most respondents' felt neutral/mixed or negative about smells in the lab, and mostly neutral about sounds. Following survey 2, the only significant change in emotional response was in relation to sounds within the anatomy lab, with a reduction in the proportion of students who found sounds to be a negative source of emotion (11% vs. 1%; *p* = 0.002).

**FIGURE 6:**
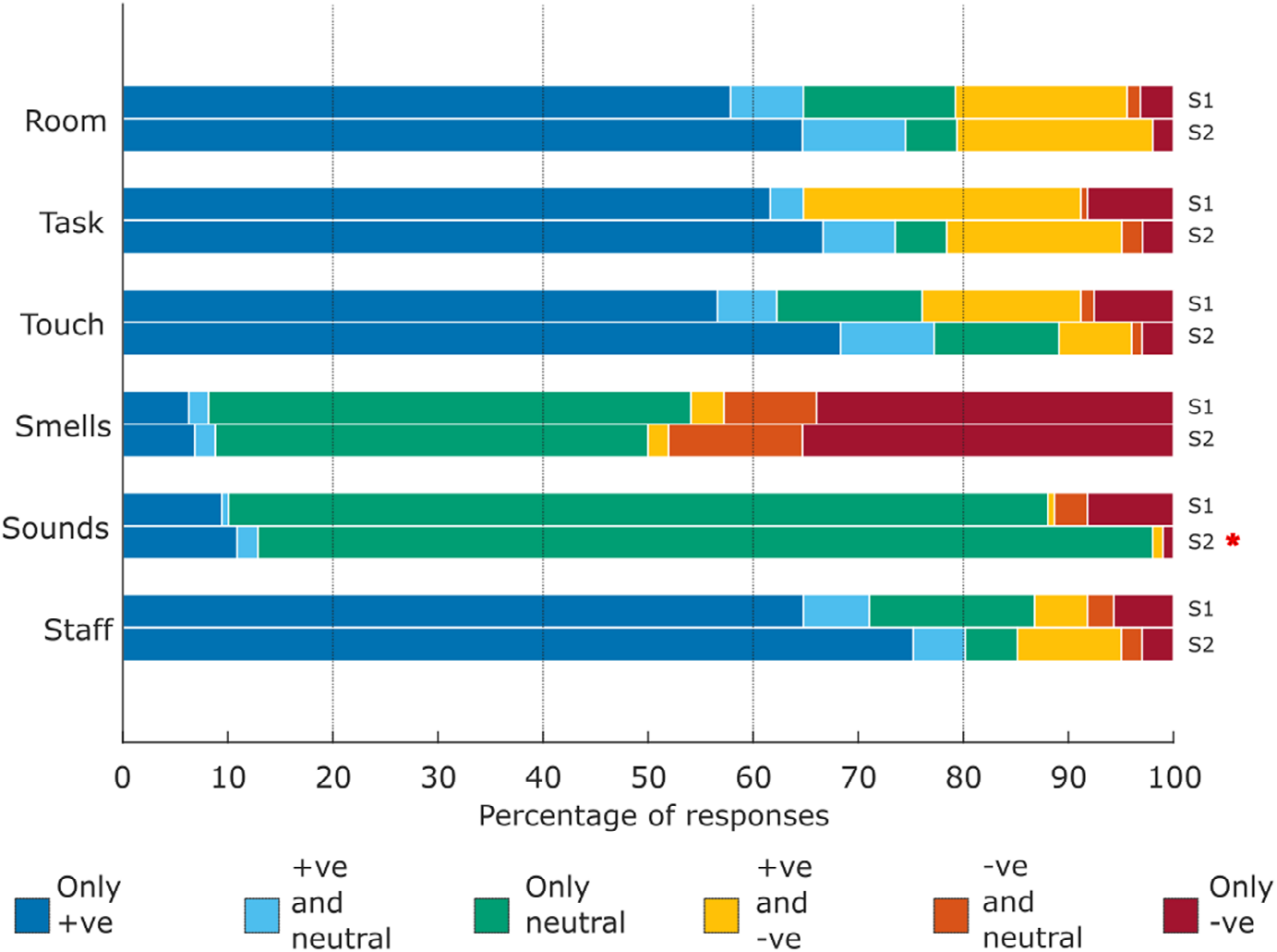
Individual students' selection of emotions by aspect of body donor encounter, for Survey 1 (S1; prior to first practical session) and Survey 2 (S2; after several months' teaching). Asterisks indicate statistically significant changes in proportions of individuals selecting negative feelings between S1 and S2.

#### Specimen type

When considering specimen type and species in Survey 1 (Figure 7), students' initial feelings about practical sessions involving all specimens were mostly positive. However, significantly more respondents felt mostly or wholly negative about sessions involving whole recognisable animals compared to isolated parts—be they small animals (e.g., dogs and cats; *p* = 0.0007), farm animals (e.g., sheep, cattle, pigs; *p* = 0.02), or equine (*p* = 0.002). There was no statistically significant difference in the proportion of respondents feeling negatively regarding the different species (e.g., farm animals vs. small animals).

**FIGURE 7:**
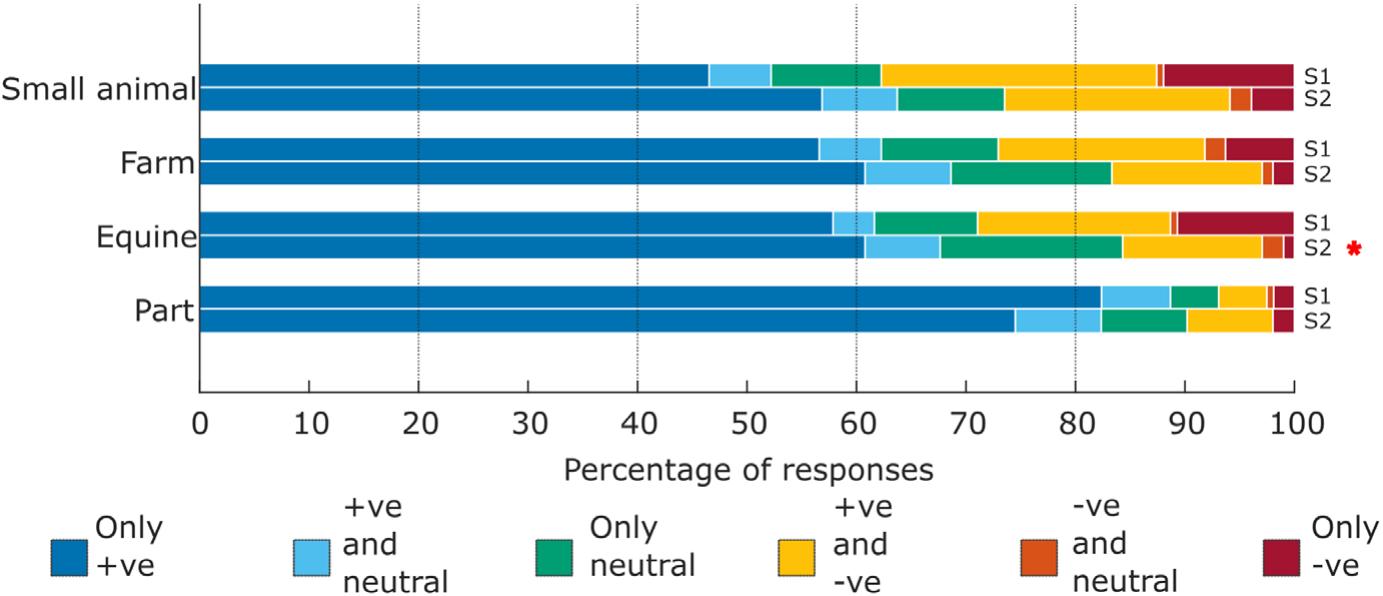
Individual students' selection of emotions by specimen type and species, for Survey 1 (S1; prior to first practical session) and Survey 2 (S2; after several months' teaching). Asterisks indicate statistically significant changes in proportions of individuals selecting mostly negative feelings between S1 and S2.

Following Survey 2, whilst there were few significant changes in emotional response to specific specimens and species, significantly fewer Survey 2 participants reported mostly negative emotions associated with equine bodies (11% vs. 3%; *p* = 0.02).

### Hypothesis 3: Cadavers, emotions, and the broader learning experience

The majority of students felt their learning was positively impacted by experiences with body donors, both in anticipation and on the follow‐up survey (Figure 8), suggesting that emotional responses around cadavers were generally helpful, rather than harmful. Regarding the use of dead animal bodies in veterinary education, almost all students (99%) agreed that their use is necessary to learn about anatomy, with the proviso that animal sourcing was an important ethical consideration (e.g., donated, purchased, euthanised for the purpose).

**FIGURE 8:**
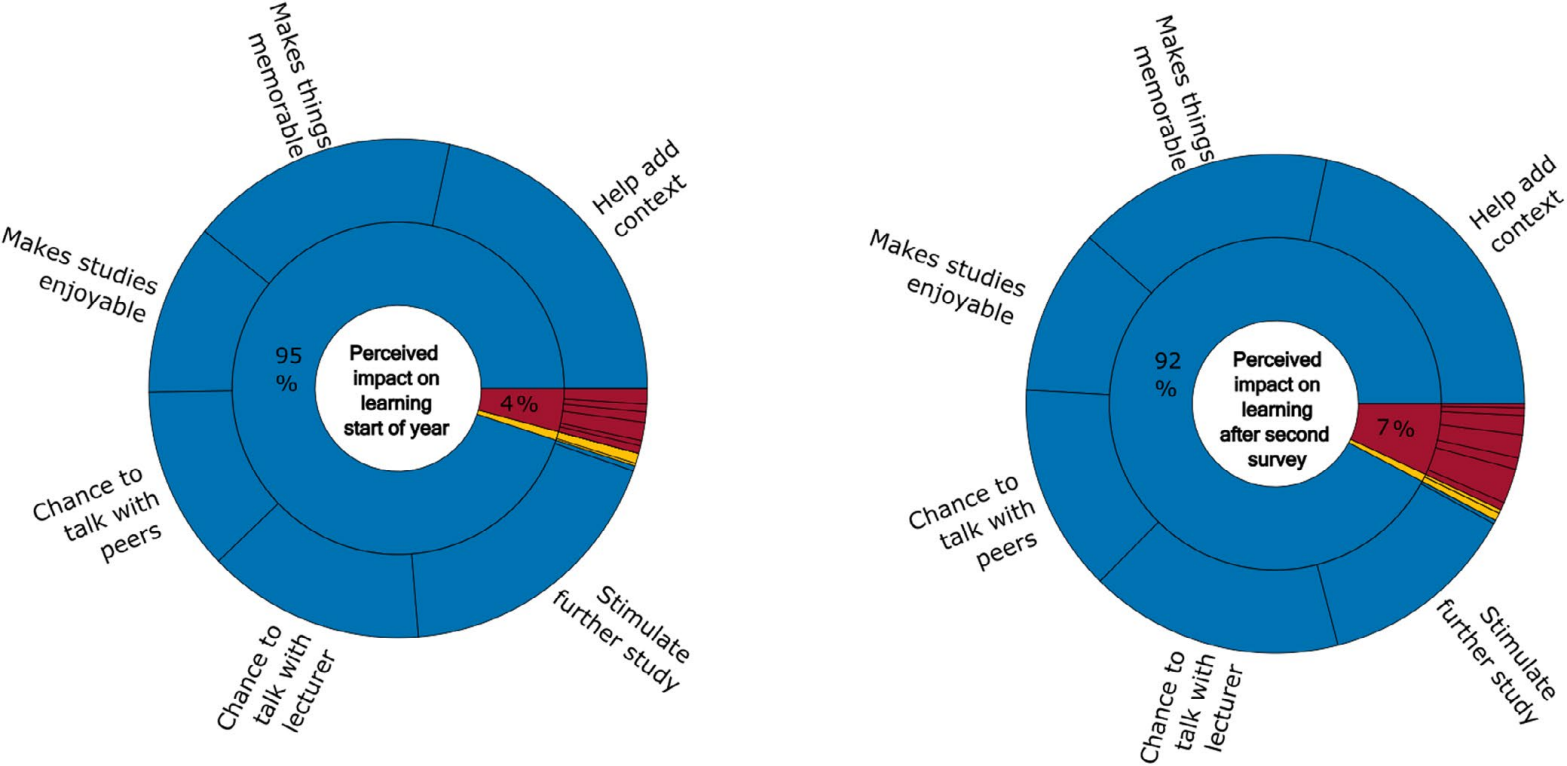
Student selection of impacts on learning.

Respondents most frequently anticipated that the impact on their studies would be to ‘Help add context’, ‘Stimulate further study’, and ‘Make things memorable’. These impacts were similar on follow‐up, with slightly more respondents choosing ‘Chance to talk with lecturer’ at the later stage. Many students elaborated on the benefits in free‐text comments, forming three key themes: academic benefits, practical benefits, and interpersonal benefits (Figure 9).

**FIGURE 9:**
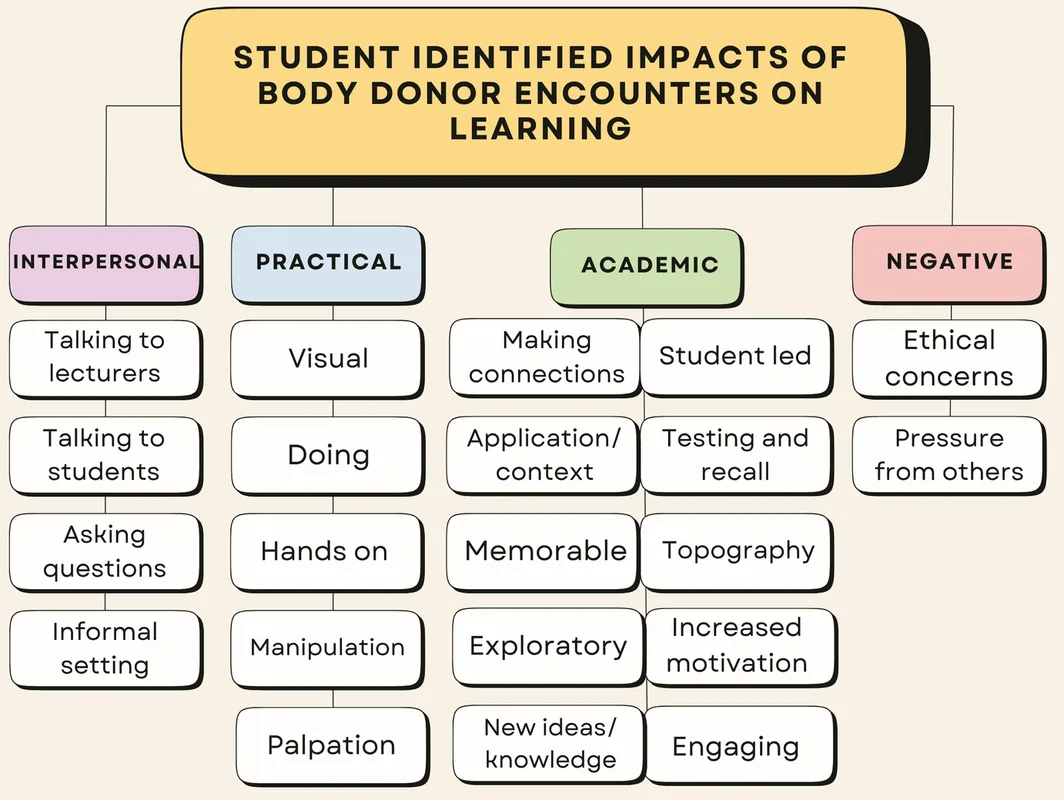
Thematic map outlining themes and codes within, with respect to student perceptions of the impact of practicals with body donors on their learning.

Many academic benefits were mentioned by students, who felt that body donors helped them to make connections between different facets of their learning, apply their learning to a relevant context, aid them with memory and recall, and provided them the opportunity to explore new ideas and acquire new knowledge. Students commented positively about the exploratory student‐led nature of dissections. They also commented that body‐donor‐based teaching was engaging and increased their motivation to learn.

Because you can see things differently from how they look in a textbook and also finding things yourself makes them more memorable and confirms what you thought you knew and corrects what you thought wrong etc. [ID S219]

They take abstract concepts from lecture and put them into the physical world where I can see and understand better [ID S211]

Practical benefits brought about by teaching with body donors included the enhanced visual nature of these sessions, the hands‐on element, including manipulation and palpation of bodies, and more generally the active learning component (‘doing’) which was felt to be beneficial to students.

I think seeing things in person and moving anatomy around with my hands is helpful [ID S245]

In high numbers, students commented on the interpersonal benefits of practical classes with body donors, including the benefit of being able to talk to and pose questions to staff, as well as to discuss topics with their peers. Students cited the informal setting as being important for their learning.

I feel like it's a chance to talk to the lecturers in a more relaxed setting and [I'm] able to talk to other people and learn about their knowledge to gain a better understanding. [ID S210]

Where students noted negative impacts on their learning, they commented about specific issues that they felt impacted them significantly enough that they could not sufficiently apply themselves to learning within the sessions. These were principally ethical concerns the students held over the use of body donor material, or the sense of being pressured by others, either staff or peers.

Sometimes some peers make me feel a bit bad/like a know it all if I know the answers. So sometimes I don't say anything since they get annoyed and I already don't have lots of friends. [ID S296]

## DISCUSSION

This study investigated how veterinary students feel about the first encounters with animal body donors in their veterinary education and how this changes during their first year. We aimed to find out whether these encounters are a source of negative emotion or stress, which factors most provoked students to experience negative emotion, and to use this information to inform how educators could improve our students' learning and experiences. We anticipate the impact of this study on curriculum design at several levels. Quantifying body‐donor‐based stressors in veterinary students is a necessary first step in implementing inclusive teaching strategies. Once the scale of any issue has been measured, evidence‐based strategies can be developed to overcome it, but institutions cannot support participation of those students who may struggle with body donor dissection if we are not aware that they exist in the first place.

### Students feel mostly positive about body donors, with little emotional change over time

Most students in this study felt positively about the idea of working with animal body donors for the first time in their veterinary education, echoing the findings of Terrado et al.^6^, and almost all considered learning with body donors to be necessary and valuable. Students cited the importance of tactile, discovery‐based learning facilitated by the body donors, supporting our hypothesis that students would feel that practical sessions involving body donors improved their educational experience. We found that most students had some form of prior contact with dead animal bodies, much of this due to the veterinary course requirements for pre‐application work experience in veterinary practices and/or farms, where encountering dead animal bodies is not unusual. We speculate that their prior experience with animal bodies may explain why relatively few students felt only negative emotion about the idea of working with animal body donors for the first time at vet school, although this correlation was not statistically supported by a prior study examining dog body donors.^6^ Unfortunately, we could not test the correlation between participants' prior cadaver experience and the likelihood of reporting negative emotion within this study.

There was little change in sentiment around working with animal body donors over time. Although we could not directly link responses between Survey 1 and 2, the evidence suggests that most students did not experience a dramatic change in their feelings—encouragingly, many students either remained positive or felt an improvement. The high number of students who felt initially positive about animal body donors in their education is likely a key reason why little change was seen with time. For the small number of students whose feelings did change, the vast majority reported this occurring within several classes—for some, within one class—as students became more familiar with the anatomy lab, and experienced first‐hand how body donors were respectfully treated and how they can assist learning. The timeline of change in veterinary students partially supports our hypothesis that negative emotions would exhibit a trajectory of rapid resolution following the initial encounter period, and mirrors the trajectory of rapid resolution observed in most medical students^1^ and in the only other study examining veterinary students, after a single class.^6^

A minority of students said that their feelings took longer to change. Additionally, it remains that all students who participated in the study may later experience further changes to their emotional responses, if their ongoing experiences further impact their feelings beyond the one‐semester time period considered within this study. Emotional change and professional distancing are likely complex, ongoing processes. For example, by Survey 2, the majority of students regarded body donors as primarily a learning tool, but the perception was not binary—many students thought about the animal's owner, final moments, and were upset when remembering the animal was once alive. There are three principal places within a veterinary curriculum where students will encounter death and animal bodies—the dissection room, during postmortem examination in the pathology laboratory, and during clinical experiences in relation to patient death and euthanasia.^24^ Therefore, student experiences with animal death will be omnipresent and evolving throughout their education and professional careers. Veterinary education that enables understanding and acceptance of the emotional responses associated with death and dying can assist with effective communication, support, and coping during this process.^25^ Experiences of dead animal bodies in the early curriculum are therefore likely to be formative, if well supported, in allowing students to prepare for later experiences in the clinical environment, through acknowledgement and reflection on their emotions, values, and beliefs.^11^, ^26^

### However, body donors, particularly some aspects, are a source of negative emotion for some students

Although most students felt positively about animal body donors, around a third experienced a mixture of positive and negative emotions, and a small number of students experienced solely negative emotions. Our hypothesis that body donor encounters would be a source of negative emotion for first‐year veterinary students is therefore partially supported. It should be noted that approximately one in ten students reported their emotional outlook to have deteriorated following contact with body donors. The magnitude to which these emotions impact the students is unclear; however, it remains a possibility that student wellbeing and learning could be impacted by experiences with body donors. Supportive conversations regarding body donors and feelings they elicit may be important to support student mental health and the development of resilience strategies. The latter are particularly important within the veterinary profession, in which veterinarians have an increased risk of suicide and mental health challenges compared with the general population.^27^ Early identification of stressors, increased emotional guidance, and development of adaptive coping skills may specifically benefit veterinary students not only in the immediacy of their first practical sessions, but throughout their professional veterinary careers. Academic and support staff should be aware that a very small proportion of students may experience deterioration of emotional well‐being after contact with body donors.

We hypothesised that negative emotional reactions would be more common for dissection classes compared with other types of body donor encounter (prosections, skeletal specimens), and for whole recognisable animals compared with isolated body parts or tracts. This hypothesis was supported. Our qualitative findings suggest that greater emotional response towards dissection‐style classes may be due to student perceptions of time inefficiency or potential for failure, as they must discover and dissect structures themselves.^28^, ^29^ However, the more active role that students must take in dissection may also go hand‐in‐hand with greater potential for learning, including appreciation of 3D relationships and teamworking, compared with prosection‐based classes or alternative anatomy resources such as videos/photos.^30^ The observed decreased negative emotion associated with ex‐situ parts may be because these are less clearly ‘animal bodies’: an organ or isolated limb may be easier to view as a learning tool, compared with a whole animal, or perhaps easier to dissociate with the idea of an animal. This mirrors the coping behaviours reported in students in clinical years of a veterinary curriculum who may detach/distance themselves from animals to cope with the emotional distress they expect to encounter in veterinary work.^8^ An alternative explanation may be that classes that involve ex‐situ parts are also correlated with less active dissection by the student, triggering lesser sensory/emotional responses.

We hypothesised that negative emotions would be more associated with companion animal species (such as dogs, cats, and horses) compared with livestock species. Unexpectedly, there did not appear to be a species difference, with small animals, farm animals, and horses prompting statistically similar reports of negative emotions in this study. This finding is surprising and conflicts with that of Herzog et al.^8^, who suggested students had more concerns about interaction with small animal body donors. The profile of veterinary students and the profession has changed somewhat since those somewhat historical qualitative interviews took place, with today's profession having a greater emphasis on companion animal practice and fewer students entering the profession from a farm background.^31^ ^,^ ^32^ This changing exposure to a broader species base, coupled with the diversification of the veterinary student population, may explain this unexpected finding.

When considering which specific factors associated with body donor use saw the most impactful change in student emotions over time, only a decrease in negative feelings about equine specimens and about sounds in the dissection room were statistically significant, given our sample size and robust corrections for multiple comparisons. One free‐text comment mentioned the student had never worked with dead horses, but otherwise, the comments did not make clear which aspects of equine bodies or the anatomy laboratory sounds the respondents were initially anticipating negatively. The most populous subcategories of negative feelings were ‘apprehensive’ and ‘anxious’. It may be that students were generally worried about the unknown (including unknown environmental aspects of the room, such as sound), but most were later reassured upon exposure to them.

### Student support and implications for practice

From our study, there are some important lessons we feel can be learned and actioned upon, to improve students' first (and subsequent) encounters with dead animal bodies and body donors. Negative emotions regarding body donors are not uncommon, and whilst arguably these feelings are appropriate and/or necessary for professional development, we should ensure that experiences with bodies are included in curricula for considered pedagogical reasons, not merely due to convenience or tradition. Pedagogical rationale should be made clear to students through an explicit introduction to the use of body donors, which addresses common points of concern raised in this study by students. For example, the rigorous diligence and efforts that institutions undertake in order to ensure ethical sourcing of material^33^ could be made more explicit to students, as supported by some free text comments in this study and elsewhere.^16^, ^34^ ^,^ ^35^ Open discussion before or at the start of teaching would be valuable to students who have concerns, for those who may not feel able to initiate such a conversation, and in fact, open ethical discussions are important for all students in developing professional decision‐making and reasoning skills, and their own moral and ethical positions. Anatomy teaching has a fundamental role in supporting the development of professional skills such as these within veterinary students.^19^, ^36^ Staff must, however, also remain alert to the (rare) instances where body donors may cause an extreme negative emotional reaction in students.

The teaching environment is important: students comment that exposure and familiarisation with the room and body donors help alleviate negative emotions. Familiarisation comes naturally as the course progresses, but offering additional introductory sessions in the anatomy lab prior to the formal start of teaching could be valuable for some students. Some institutions allow the anatomy laboratory to be used for extracurricular activities (e.g., drawing club, anatomy club), which could also help de‐mystify the space. If found to be effective, such acclimation could be beneficial for similarly intimidating clinical spaces later in the curriculum (e.g., post‐mortem room, operating theater, consulting room).

The psychological burden to make the best use of the body donor and the limited time in the anatomy lab was featured as a concern for participants in this study. Teaching time in the laboratory with body donors has been universally reduced in medical and veterinary courses, and this appears to be reflected in the pressures students report. Encounters with body donors have been suggested to take on heightened significance for students in curricula where such experiences are infrequent^11^ when compared to more traditional curricula. More timetabled or extracurricular sessions could therefore be beneficial. Where this is not possible, acknowledging resources, both human and body donor, are limited, we as educators must ensure that students are able to make the best use of their time in the anatomy laboratory; specifically, that negative emotional states are not further harming the limited practical teaching time available to students. Provision of supporting or preparatory materials can be helpful here; their value relies on students' time and ability to engage with the supplementary teaching material, but has been shown to be effective in improving student performance^37^ and may also support student wellbeing/emotional preparedness in this context.^6^

Replacement or reduction of body‐donor‐based teaching in some curricula has been deployed as a teaching philosophy, predominantly driven by student numbers, high cost of developing and maintaining body donor resources and facilities, and to adjust in circumstances such as the COVID‐19 pandemic.^6^, ^38^, ^39^, ^40^ ^,^ ^41^ The feelings and opinions of students about body donor use here can contribute additional insights into the rationale and debate on reduction or replacement of body‐donor‐based teaching. Primarily, factors reported here by students as important to their experience, such as self‐discovery and tactile elements, can be difficult to replicate with other modalities. Students also acknowledged indirect interpersonal benefits of the teaching context body donors are used in, besides the direct academic benefits and practical skills gained from the bodies themselves. These, too, require careful planning to replicate if body‐donor‐based teaching is replaced, and may be more easily overlooked. For example, alternatives to body donors may be more likely to involve screen‐based resources (e.g., computer models, photos, videos) and lend themselves more to individual/lone working. Students may lose out on the indirect benefits of dissection, such as unstructured exploratory time and the ‘side‐by‐side’, task‐oriented nature of classes that can encourage conversation with lecturers and peers. Dissection also lends itself to division of roles (cutter, scribe, holder, researcher, question‐asker) and team working that mimics the surgical or veterinary team working environment. The latter has been encouraged by some curricula.^42^ Careful consideration of the interpersonal and social elements, as well as more tangible academic and practical elements, is needed if body‐donor‐based teaching were replaced.^43^

### Limitations and future research

We recognise that our study does have some limitations. Firstly, this is a small study involving only three veterinary institutions in the UK. As such, the sample size restricted some analyses and likely influenced the non‐statistical significance of some comparisons. Although statistical tests have been used to infer significance here, we must remember that each response represents a real person, so small numbers of negative responses, even if ‘statistically insignificant’, are still important to acknowledge, to ensure that the well‐being and support needs of all students are catered for. Secondly, fewer students participated in the second survey, and whilst we have treated the respondents as representative of their cohort, we do not know if there are reasons why students may be lost to follow‐up that could be relevant to their feelings on body donors (e.g., students who have become disenfranchised). Thirdly, the nature of responses may be sensitive to priming, i.e., the prior information students have received (including information within the survey itself, such as support signposting or questions probing hypothetical body donor provenance). In particular, students' views on the necessity of body donors could reflect a bias of the study population: all students recruited for this study attended universities where the curriculum included at least some traditional cadaver‐based anatomy classes. Learning via real animal dead bodies is often pitched as invaluable for veterinary students, due to skills gained with tools and tissue handling, its automatic inclusion of anatomical variants, and the need for surgeons not just to identify but also find and expose anatomical structures.^44^ Students at the participating institutions may be more likely to have ‘bought in’ to this rationale, or made their choice of university based on the curriculum's inclusion of more practical classes involving real animal bodies.

It is important not to over‐correct on the basis of smaller studies, such as ours and Terrado et al.^6^ Future research is important in confirming whether our findings are reproducible for larger study populations (e.g., other veterinary schools) and cultural contexts. Follow‐up research over a longer timescale, and inclusion of graduated veterinarian's opinions, would also be valuable in understanding the longer‐term impact of body donor encounters on learning and mental health. Some negative emotions can have their place in learning (e.g., as eustress, or drivers^15^), and we should not seek to eliminate them unless they are harmful. Other negative emotions, such as sadness, are important to feel and reflect upon, in order to appropriately recognise the gift of learning that the dead impart to us. Well‐supported encounters with body donors are an opportunity for the development of practical skills, academic learning, professional identity, emotional discovery, and resilience.

## CONCLUSIONS

Most students feel positively about their first encounters with animal body donors in veterinary school, and remain stable and positive following their learning experiences with animal bodies throughout the first year. Almost all (99%) feel that the use of body donors is vital for learning about anatomy, and that learning with body donors improves their veterinary education. However, body donor encounter is a source of solely negative emotion for a small proportion of students. For most students for whom emotional response was not initially positive, it took several classes with body donors before their feelings changed. Classes that require dissection or that involve whole recognisable animals prompt more students to feel negatively; however, type of species encountered does not influence emotional response. The findings suggest that whilst animal body donors are an important part of veterinary anatomy education, some students may require early support to promote eustress rather than distress around body donor use. We provide recommendations for anatomy educators and curriculum leaders on how best to support students during early encounters with dead animal bodies in veterinary programmes.

## AUTHOR CONTRIBUTIONS

**S. Regnault:** Conceptualization; formal analysis; investigation; project administration; visualization; writing – original draft; writing – review and editing. **C. Basu:** Investigation; project administration; visualization; writing – review and editing. **S. Channon:** Investigation; project administration; formal analysis; funding acquisition; writing – original draft; writing – review and editing.

## FUNDING INFORMATION

The study received no external funding.

## CONFLICT OF INTEREST STATEMENT

The authors have no conflict of interest to declare.

## ETHICS STATEMENT

Ethical approval was granted by Aberystwyth University, The Royal Veterinary College, and The University of Surrey School of Veterinary Medicine.

## Supporting information


**Data S1.** Survey Questions

## Data Availability

Data is available on request to the authors.
