# Supplementary material for: Emotional responses of veterinary students to first encounters with animal body donors
Source: Anat Sci Educ. 2026 Jul 14;19(10):1633–48. doi: 10.1002/ase.70291 (PMC13654053; doi:10.1002/ase.70291)
Supplement: Supplementary file 1 [file ASE-19-1633-s001.docx]

Survey 1

In the questions below, please tick the feelings that apply. You can tick more than one feeling, and you can specify something else if it isn’t listed.

1) How do you feel about being in the dissection room and working with animal cadavers (dead bodies) for the first time?

| Positive | Neutral | Negative |
| --- | --- | --- |
| ⃝ Looking-forward to  ⃝ Interested  ⃝ Supported  ⃝ Confident  ⃝ Excited  ⃝ Fun / enjoyable  ⃝ Other: | ⃝ Not really thought about it  ⃝ Fine / ok  ⃝ Don’t care  ⃝ Don’t think its important  ⃝ Other: | ⃝ Isolated / lonely  ⃝ Apprehensive  ⃝ Terrified  ⃝ Anxious  ⃝ Confused  ⃝ Sick / disgusted  ⃝ Other: |

2) Please suggest why you feel this way about working with cadavers in the dissection room.

3) Have you had any prior experience with cadavers, either in your personal life (e.g. deceased pet), on a farm, or in a veterinary setting?

| ⃝ Yes  Please state what kind of experience: | ⃝ No |
| --- | --- |

4) How do you feel about being in the dissection room itself?

| Positive | Neutral | Negative |
| --- | --- | --- |
| ⃝ Looking-forward to  ⃝ Interested  ⃝ Supported  ⃝ Confident  ⃝ Excited  ⃝ Fun / enjoyable  ⃝ Other: | ⃝ Not really thought about it  ⃝ Fine / ok  ⃝ Don’t care  ⃝ Don’t think its important  ⃝ Other: | ⃝ Isolated / lonely  ⃝ Apprehensive  ⃝ Terrified  ⃝ Anxious  ⃝ Confused  ⃝ Sick / disgusted  ⃝ Other: |

5) How do you feel about practicals involving bones and skeletons?

| Positive | Neutral | Negative |
| --- | --- | --- |
| ⃝ Looking-forward to  ⃝ Interested  ⃝ Supported  ⃝ Confident  ⃝ Excited  ⃝ Fun / enjoyable  ⃝ Other: | ⃝ Not really thought about it  ⃝ Fine / ok  ⃝ Don’t care  ⃝ Don’t think its important  ⃝ Other: | ⃝ Isolated / lonely  ⃝ Apprehensive  ⃝ Terrified  ⃝ Anxious  ⃝ Confused  ⃝ Sick / disgusted  ⃝ Other: |

6) How do you feel about practicals involving prosections (where cadavers are dissected ahead of time by an academic, ready for students to view)?

| Positive | Neutral | Negative |
| --- | --- | --- |
| ⃝ Looking-forward to  ⃝ Interested  ⃝ Supported  ⃝ Confident  ⃝ Excited  ⃝ Fun / enjoyable  ⃝ Other: | ⃝ Not really thought about it  ⃝ Fine / ok  ⃝ Don’t care  ⃝ Don’t think its important  ⃝ Other: | ⃝ Isolated / lonely  ⃝ Apprehensive  ⃝ Terrified  ⃝ Anxious  ⃝ Confused  ⃝ Sick / disgusted  ⃝ Other: |

7) How do you feel about practicals involving dissection (where students perform the dissection)?

| Positive | Neutral | Negative |
| --- | --- | --- |
| ⃝ Looking-forward to  ⃝ Interested  ⃝ Supported  ⃝ Confident  ⃝ Excited  ⃝ Fun / enjoyable  ⃝ Other: | ⃝ Not really thought about it  ⃝ Fine / ok  ⃝ Don’t care  ⃝ Don’t think its important  ⃝ Other: | ⃝ Isolated / lonely  ⃝ Apprehensive  ⃝ Terrified  ⃝ Anxious  ⃝ Confused  ⃝ Sick / disgusted  ⃝ Other: |

8) How do you feel about practicals involving whole recognisable small animal cadavers (e.g. dogs and cats)?

| Positive | Neutral | Negative |
| --- | --- | --- |
| ⃝ Looking-forward to  ⃝ Interested  ⃝ Supported  ⃝ Confident  ⃝ Excited  ⃝ Fun / enjoyable  ⃝ Other: | ⃝ Not really thought about it  ⃝ Fine / ok  ⃝ Don’t care  ⃝ Don’t think its important  ⃝ Other: | ⃝ Isolated / lonely  ⃝ Apprehensive  ⃝ Terrified  ⃝ Anxious  ⃝ Confused  ⃝ Sick / disgusted  ⃝ Other: |

9) How do you feel about practicals involving whole recognisable farm animal cadavers (e.g. sheep and pigs)?

| Positive | Neutral | Negative |
| --- | --- | --- |
| ⃝ Looking-forward to  ⃝ Interested  ⃝ Supported  ⃝ Confident  ⃝ Excited  ⃝ Fun / enjoyable  ⃝ Other: | ⃝ Not really thought about it  ⃝ Fine / ok  ⃝ Don’t care  ⃝ Don’t think its important  ⃝ Other: | ⃝ Isolated / lonely  ⃝ Apprehensive  ⃝ Terrified  ⃝ Anxious  ⃝ Confused  ⃝ Sick / disgusted  ⃝ Other: |

10) How do you feel about practicals involving whole recognisable horse cadavers?

| Positive | Neutral | Negative |
| --- | --- | --- |
| ⃝ Looking-forward to  ⃝ Interested  ⃝ Supported  ⃝ Confident  ⃝ Excited  ⃝ Fun / enjoyable  ⃝ Other: | ⃝ Not really thought about it  ⃝ Fine / ok  ⃝ Don’t care  ⃝ Don’t think its important  ⃝ Other: | ⃝ Isolated / lonely  ⃝ Apprehensive  ⃝ Terrified  ⃝ Anxious  ⃝ Confused  ⃝ Sick / disgusted  ⃝ Other: |

11) How do you feel about practicals involving isolated parts of animals (e.g. isolated organs or tracts)?

| Positive | Neutral | Negative |
| --- | --- | --- |
| ⃝ Looking-forward to  ⃝ Interested  ⃝ Supported  ⃝ Confident  ⃝ Excited  ⃝ Fun / enjoyable  ⃝ Other: | ⃝ Not really thought about it  ⃝ Fine / ok  ⃝ Don’t care  ⃝ Don’t think its important  ⃝ Other: | ⃝ Isolated / lonely  ⃝ Apprehensive  ⃝ Terrified  ⃝ Anxious  ⃝ Confused  ⃝ Sick / disgusted  ⃝ Other: |

12) How do you feel about performing dissection (cutting and identifying anatomical parts)?

| Positive | Neutral | Negative |
| --- | --- | --- |
| ⃝ Looking-forward to  ⃝ Interested  ⃝ Supported  ⃝ Confident  ⃝ Excited  ⃝ Fun / enjoyable  ⃝ Other: | ⃝ Not really thought about it  ⃝ Fine / ok  ⃝ Don’t care  ⃝ Don’t think its important  ⃝ Other: | ⃝ Isolated / lonely  ⃝ Apprehensive  ⃝ Terrified  ⃝ Anxious  ⃝ Confused  ⃝ Sick / disgusted  ⃝ Other: |

13) How do you feel about touching and manipulating animal cadavers?

| Positive | Neutral | Negative |
| --- | --- | --- |
| ⃝ Looking-forward to  ⃝ Interested  ⃝ Supported  ⃝ Confident  ⃝ Excited  ⃝ Fun / enjoyable  ⃝ Other: | ⃝ Not really thought about it  ⃝ Fine / ok  ⃝ Don’t care  ⃝ Don’t think its important  ⃝ Other: | ⃝ Isolated / lonely  ⃝ Apprehensive  ⃝ Terrified  ⃝ Anxious  ⃝ Confused  ⃝ Sick / disgusted  ⃝ Other: |

14) How do you feel about the smells that there may be in the dissection room?

| Positive | Neutral | Negative |
| --- | --- | --- |
| ⃝ Looking-forward to  ⃝ Interested  ⃝ Supported  ⃝ Confident  ⃝ Excited  ⃝ Fun / enjoyable  ⃝ Other: | ⃝ Not really thought about it  ⃝ Fine / ok  ⃝ Don’t care  ⃝ Don’t think its important  ⃝ Other: | ⃝ Isolated / lonely  ⃝ Apprehensive  ⃝ Terrified  ⃝ Anxious  ⃝ Confused  ⃝ Sick / disgusted  ⃝ Other: |

15) How do you feel about the sounds that there may be in the dissection room?

| Positive | Neutral | Negative |
| --- | --- | --- |
| ⃝ Looking-forward to  ⃝ Interested  ⃝ Supported  ⃝ Confident  ⃝ Excited  ⃝ Fun / enjoyable  ⃝ Other: | ⃝ Not really thought about it  ⃝ Fine / ok  ⃝ Don’t care  ⃝ Don’t think its important  ⃝ Other: | ⃝ Isolated / lonely  ⃝ Apprehensive  ⃝ Terrified  ⃝ Anxious  ⃝ Confused  ⃝ Sick / disgusted  ⃝ Other: |

16) How do you feel about interacting with the teacher (lecturer or academic) in the dissection room?

| Positive | Neutral | Negative |
| --- | --- | --- |
| ⃝ Looking-forward to  ⃝ Interested  ⃝ Supported  ⃝ Confident  ⃝ Excited  ⃝ Fun / enjoyable  ⃝ Other: | ⃝ Not really thought about it  ⃝ Fine / ok  ⃝ Don’t care  ⃝ Don’t think its important  ⃝ Other: | ⃝ Isolated / lonely  ⃝ Apprehensive  ⃝ Terrified  ⃝ Anxious  ⃝ Confused  ⃝ Sick / disgusted  ⃝ Other: |

17) How do you think your feelings about cadavers or the dissection room impacts your studies?

| Positive | Neutral | Negative |
| --- | --- | --- |
| ⃝ Helps add context  ⃝ Makes things memorable  ⃝ Makes studies enjoyable  ⃝ Chance to talk with peers  ⃝ Chance to talk with teacher  ⃝ Stimulates further study  ⃝ Other: | ⃝ It doesn’t affect my studies  ⃝ Fine / ok  ⃝ Don’t care  ⃝ Don’t think its important  ⃝ Other: | ⃝ Won’t be able to focus  ⃝ Won’t recall knowledge  ⃝ Won’t enjoy class  ⃝ Worried outside of class  ⃝ Can’t relate to peers  ⃝ Won’t want to attend class  ⃝ Other: |

18) Considering your responses to Q3 – 16, is there anything else you like to tell us about your emotions in relation to cadaver use? Please state here:

19) Considering your responses to this survey, please provide any further information that will help us understand your views and feelings about using cadavers in veterinary anatomy teaching

Survey 2

1) How did you feel about being in the dissection room and working with animal cadavers (dead bodies) for the first time?

⃝ Generally positive

⃝ Generally negative

⃝ Generally neutral

⃝ A mix of positive and negative for different aspects

2) How do you feel now about being in the dissection room itself?

| Positive | Neutral | Negative |
| --- | --- | --- |
| ⃝ Look forward to it  ⃝ Interested  ⃝ Supported  ⃝ Confident  ⃝ Excited  ⃝ Fun / enjoyable  ⃝ Other: | ⃝ Don’t really think about it  ⃝ Fine / ok  ⃝ Don’t care  ⃝ Don’t think its important  ⃝ Other: | ⃝ Isolated / lonely  ⃝ Apprehensive  ⃝ Terrified  ⃝ Anxious  ⃝ Confused  ⃝ Sick / disgusted  ⃝ Other: |

3) If your feelings about being in the dissection room itself changed throughout the semester, how long did this take?

⃝ My feelings didn’t change

⃝ My feelings generally changed within one class

⃝ My feelings generally changed after one class

⃝ My feelings generally changed after a few classes

⃝ My feelings changed by the end of Semester 1

4) Suggest why your feelings about cadaver use and/or being in the dissection room changed (or did not change)

5) How do you feel now about practicals involving bones and skeletons?

| Positive | Neutral | Negative |
| --- | --- | --- |
| ⃝ Look forward to it  ⃝ Interested  ⃝ Supported  ⃝ Confident  ⃝ Excited  ⃝ Fun / enjoyable  ⃝ Other: | ⃝ Don’t really think about it  ⃝ Fine / ok  ⃝ Don’t care  ⃝ Don’t think its important  ⃝ Other: | ⃝ Isolated / lonely  ⃝ Apprehensive  ⃝ Terrified  ⃝ Anxious  ⃝ Confused  ⃝ Sick / disgusted  ⃝ Other: |

6) How do you feel now about practicals involving prosections (where cadavers are dissected ahead of time by an academic, ready for students to view)?

| Positive | Neutral | Negative |
| --- | --- | --- |
| ⃝ Look forward to it  ⃝ Interested  ⃝ Supported  ⃝ Confident  ⃝ Excited  ⃝ Fun / enjoyable  ⃝ Other: | ⃝ Don’t really think about it  ⃝ Fine / ok  ⃝ Don’t care  ⃝ Don’t think its important  ⃝ Other: | ⃝ Isolated / lonely  ⃝ Apprehensive  ⃝ Terrified  ⃝ Anxious  ⃝ Confused  ⃝ Sick / disgusted  ⃝ Other: |

7) How do you feel now about practicals involving dissection (where students perform the dissection)?

| Positive | Neutral | Negative |
| --- | --- | --- |
| ⃝ Look forward to it  ⃝ Interested  ⃝ Supported  ⃝ Confident  ⃝ Excited  ⃝ Fun / enjoyable  ⃝ Other: | ⃝ Don’t really think about it  ⃝ Fine / ok  ⃝ Don’t care  ⃝ Don’t think its important  ⃝ Other: | ⃝ Isolated / lonely  ⃝ Apprehensive  ⃝ Terrified  ⃝ Anxious  ⃝ Confused  ⃝ Sick / disgusted  ⃝ Other: |

8) How do you feel now about practicals involving whole recognisable small animal cadavers (e.g. dogs and cats)?

| Positive | Neutral | Negative |
| --- | --- | --- |
| ⃝ Look forward to it  ⃝ Interested  ⃝ Supported  ⃝ Confident  ⃝ Excited  ⃝ Fun / enjoyable  ⃝ Other: | ⃝ Don’t really think about it  ⃝ Fine / ok  ⃝ Don’t care  ⃝ Don’t think its important  ⃝ Other: | ⃝ Isolated / lonely  ⃝ Apprehensive  ⃝ Terrified  ⃝ Anxious  ⃝ Confused  ⃝ Sick / disgusted  ⃝ Other: |

9) How do you feel now about practicals involving whole recognisable farm animal cadavers (e.g. sheep and pigs)?

| Positive | Neutral | Negative |
| --- | --- | --- |
| ⃝ Look forward to it  ⃝ Interested  ⃝ Supported  ⃝ Confident  ⃝ Excited  ⃝ Fun / enjoyable  ⃝ Other: | ⃝ Don’t really think about it  ⃝ Fine / ok  ⃝ Don’t care  ⃝ Don’t think its important  ⃝ Other: | ⃝ Isolated / lonely  ⃝ Apprehensive  ⃝ Terrified  ⃝ Anxious  ⃝ Confused  ⃝ Sick / disgusted  ⃝ Other: |

10) How do you feel now about practicals involving whole recognisable horse cadavers?

| Positive | Neutral | Negative |
| --- | --- | --- |
| ⃝ Look forward to it  ⃝ Interested  ⃝ Supported  ⃝ Confident  ⃝ Excited  ⃝ Fun / enjoyable  ⃝ Other: | ⃝ Don’t really think about it  ⃝ Fine / ok  ⃝ Don’t care  ⃝ Don’t think its important  ⃝ Other: | ⃝ Isolated / lonely  ⃝ Apprehensive  ⃝ Terrified  ⃝ Anxious  ⃝ Confused  ⃝ Sick / disgusted  ⃝ Other: |

11) How do you feel now about practicals involving parts of animals (e.g. organs or tracts)?

| Positive | Neutral | Negative |
| --- | --- | --- |
| ⃝ Look forward to it  ⃝ Interested  ⃝ Supported  ⃝ Confident  ⃝ Excited  ⃝ Fun / enjoyable  ⃝ Other: | ⃝ Don’t really think about it  ⃝ Fine / ok  ⃝ Don’t care  ⃝ Don’t think its important  ⃝ Other: | ⃝ Isolated / lonely  ⃝ Apprehensive  ⃝ Terrified  ⃝ Anxious  ⃝ Confused  ⃝ Sick / disgusted  ⃝ Other: |

12) How do you feel now about performing dissection (cutting and identifying anatomical parts)?

| Positive | Neutral | Negative |
| --- | --- | --- |
| ⃝ Look forward to it  ⃝ Interested  ⃝ Supported  ⃝ Confident  ⃝ Excited  ⃝ Fun / enjoyable  ⃝ Other: | ⃝ Don’t really think about it  ⃝ Fine / ok  ⃝ Don’t care  ⃝ Don’t think its important  ⃝ Other: | ⃝ Isolated / lonely  ⃝ Apprehensive  ⃝ Terrified  ⃝ Anxious  ⃝ Confused  ⃝ Sick / disgusted  ⃝ Other: |

13) How do you feel now about touching and manipulating animal cadavers?

| Positive | Neutral | Negative |
| --- | --- | --- |
| ⃝ Look forward to it  ⃝ Interested  ⃝ Supported  ⃝ Confident  ⃝ Excited  ⃝ Fun / enjoyable  ⃝ Other: | ⃝ Don’t really think about it  ⃝ Fine / ok  ⃝ Don’t care  ⃝ Don’t think its important  ⃝ Other: | ⃝ Isolated / lonely  ⃝ Apprehensive  ⃝ Terrified  ⃝ Anxious  ⃝ Confused  ⃝ Sick / disgusted  ⃝ Other: |

14) How do you feel now about the smells in the dissection room?

| Positive | Neutral | Negative |
| --- | --- | --- |
| ⃝ Look forward to it  ⃝ Interested  ⃝ Supported  ⃝ Confident  ⃝ Excited  ⃝ Fun / enjoyable  ⃝ Other: | ⃝ Don’t really think about it  ⃝ Fine / ok  ⃝ Don’t care  ⃝ Don’t think its important  ⃝ Other: | ⃝ Isolated / lonely  ⃝ Apprehensive  ⃝ Terrified  ⃝ Anxious  ⃝ Confused  ⃝ Sick / disgusted  ⃝ Other: |

15) How do you feel now about the sounds in the dissection room?

| Positive | Neutral | Negative |
| --- | --- | --- |
| ⃝ Look forward to it  ⃝ Interested  ⃝ Supported  ⃝ Confident  ⃝ Excited  ⃝ Fun / enjoyable  ⃝ Other: | ⃝ Don’t really think about it  ⃝ Fine / ok  ⃝ Don’t care  ⃝ Don’t think its important  ⃝ Other: | ⃝ Isolated / lonely  ⃝ Apprehensive  ⃝ Terrified  ⃝ Anxious  ⃝ Confused  ⃝ Sick / disgusted  ⃝ Other: |

16) How do you feel now about interacting with the teacher (lecturer or academic) in the dissection room?

| Positive | Neutral | Negative |
| --- | --- | --- |
| ⃝ Look forward to it  ⃝ Interested  ⃝ Supported  ⃝ Confident  ⃝ Excited  ⃝ Fun / enjoyable  ⃝ Other: | ⃝ Don’t really think about it  ⃝ Fine / ok  ⃝ Don’t care  ⃝ Don’t think its important  ⃝ Other: | ⃝ Isolated / lonely  ⃝ Apprehensive  ⃝ Terrified  ⃝ Anxious  ⃝ Confused  ⃝ Sick / disgusted  ⃝ Other: |

17) If you think your feelings about any of the types of practicals or specimens have changed since the first survey, how long did it take?

⃝ My feelings didn’t change

⃝ My feelings generally changed within one class

⃝ My feelings generally changed after one class

⃝ My feelings generally changed after a few classes

⃝ My feelings changed by the end of Semester 1

18) Looking back, do you think your feelings about cadavers or the dissection room have impacted your studies?

| Positive | Neutral | Negative |
| --- | --- | --- |
| ⃝ Helped add context  ⃝ Made things memorable  ⃝ Made studies enjoyable  ⃝ Chance to talk with peers  ⃝ Chance to talk with teacher  ⃝ Stimulated further study  ⃝ Other: | ⃝ It doesn’t affect my studies  ⃝ Fine / ok  ⃝ Don’t care  ⃝ Don’t think its important  ⃝ Other: | ⃝ Not be able to focus  ⃝ Can’t recall knowledge  ⃝ Don’t enjoy class  ⃝ Worried outside of class  ⃝ Can’t relate to peers  ⃝ Don’t want to attend class  ⃝ Other: |

19) Why do you feel cadavers and/ or the dissection room have impacted your learning in this way?

20) Please indicate whether you agree or disagree with the below statements, when you used cadavers in your classes.
a) I regard the cadaver as just a learning tool

⃝ Agree	⃝ Disagree

b) I’m not upset when I remember that the cadaver was once a live animal

⃝ Agree	⃝ Disagree

c) I think about the animal’s owner

⃝ Agree	⃝ Disagree

d) I think about the animal’s final moments

⃝ Agree	⃝ Disagree

21) How do you feel about use of cadavers in veterinary medicine courses? Please select the closest option, or if none are closest please specify.
    - Use of cadavers is always ethically unjustifiable no matter how necessary
    - Use of cadavers is necessary to learn about anatomy but is difficult to justify ethically
    - Use of cadavers is necessary to learn about anatomy and ethically fine
    - Use of cadavers is necessary to learn about anatomy but the ethics depend on where the animals come from (e.g. donated, purchased, euthanised for purpose)
    - Use of cadavers is unnecessary to learn about anatomy, other methods would be just as good (photos, videos, computer models)
    - Other (please specify):

22) Is there any further information you wish to provide that will help us understand your views and feelings about using cadavers in veterinary anatomy teaching ?
